# Abscopal Brain Proteomic Changes Associated with Microbiome Alterations Induced by Gastrointestinal Acute Radiation Syndrome in Swine

**DOI:** 10.3390/ijms26178121

**Published:** 2025-08-22

**Authors:** Kathleen Hatch, Timothy S. Horseman, Babita Parajuli, Erin K. Murphy, Robert N. Cole, Robert N. O’Meally, Daniel P. Perl, David M. Burmeister, Diego Iacono

**Affiliations:** 1 DoD/USU Brain Tissue Repository & Neuropathology Program, Uniformed Services University (USU), Bethesda, MD 20814, USA; kathleen.hatch.ctr@usuhs.edu (K.H.); erin.murphy.ctr@usuhs.edu (E.K.M.); daniel.perl@usuhs.edu (D.P.P.); 2 Department of Pathology, F. Edward Hébert School of Medicine, Uniformed Services University (USU), Bethesda, MD 20814, USA; 3 The Henry M. Jackson Foundation for the Advancement of Military Medicine (HJF) Inc., Bethesda, MD 20817, USA; 4 Department of Medicine, F. Edward Hébert School of Medicine, Uniformed Services University (USU), Bethesda, MD 20814, USAbabita.parajuli.ctr@usuhs.edu (B.P.); david.burmeister@usuhs.edu (D.M.B.); 5 Armed Forces Radiobiology Research Institute, Uniformed Services University (USU), Bethesda, MD 20814, USA; 6 Mass Spectrometry and Proteomics, Department of Biological Chemistry, School of Medicine, Johns Hopkins University, Baltimore, MD 20814, USA; rcole@jhmi.edu (R.N.C.); romeally@jhmi.edu (R.N.O.); 7 Department of Neurology, F. Edward Hébert School of Medicine, Uniformed Services University (USU), Bethesda, MD 20814, USA; 8 Neuroscience Program, Department of Anatomy, Physiology & Genetics, Uniformed Services University (USU), Bethesda, MD 20814, USA

**Keywords:** gut–brain axis, pelvic irradiation, microbiomics, proteomics, frontal cortex

## Abstract

Emerging research highlights the gut microbiota’s critical role in modulating brain activity via the gut–brain axis. This study explores whether targeted gastrointestinal irradiation induces abscopal effects on the brain proteome, revealing microbiota-mediated neurobiological changes. Male Sinclair minipigs were randomized to receive either sham treatment (*n* = 6) or 8 Gy lower hemibody (gut-targeted) irradiation (*n* = 5). Over 14 days, rectal swabs were collected to monitor microbiota dynamics, followed by frontal cortex proteomic analysis. Irradiation altered gut microbiota composition, notably reducing Chlamydiae and Firmicutes phyla, while increasing Coriobacteriaceae and Acinetobacter. Proteomic analysis identified 75 differentially abundant proteins in the frontal cortex, including a significant decrease in pannexin-1 (PANX1), suggesting modulation of the NLRP3 inflammasome pathway. Functional enrichment analysis revealed immune and neurotransmission-related changes linked to microbial shifts. These results demonstrate that gut-targeted radiation can remotely affect brain protein expression, emphasizing the microbiota’s role in neuroimmune regulation and pointing to novel therapeutic opportunities in gut–brain axis disorders.

## 1. Introduction

The gut–brain axis (GBA) is one of the most complex and rapidly evolving fields in mammalian microbiology and neurobiology. It comprises a bidirectional network of anatomical and biochemical connections linking the gastrointestinal (GI) system, the enteric nervous system (ENS), and the central nervous system (CNS). These mutual connections operate both directly—via vagal afferents—and indirectly through endocrine and immune mediators. The interplay among these systems is further influenced by the vast and taxonomically diverse microbiota inhabiting the GI tract [1].

The bidirectional communication among microbes and the CNS involves specific microbial signals traversing the ENS and reaching the brain via vagal afferents and endocrine mediators, whereas, on the other side, brain-derived molecules modulate both GI and ENS functions [2]. This intricate interplay between the “*major-brain*” and the “*gut-brain*” highlights the physiological significance of host-specific microbial species. Emerging research suggests that commensal gut microbiota alterations or imbalances (dysbiosis) may contribute to the pathogenesis of neurological and psychiatric disorders [3,4], opening new perspectives on how external environmental factors and microbial communities influence brain function and structure during brain development [5].

Generally, various experimental approaches have been developed to study the effects of gut microbiota dysbiosis on the ENS and brain [6], including hemibody irradiation to induce post-radiation dysbiosis and assess potential countermeasures. However, very little is known about the possible abscopal effects of high-dose abdominal irradiation on the brain.

A previous study in a large animal (swine) demonstrated that acute gastrointestinal (GI) irradiation with a total dose of 8 Gy led to distinct microbiome alterations 14 days post-exposure [7]. Building on these findings, we aimed to investigate whether proteomic changes could occur in the brain—a non-irradiated organ—of the same animals and time point (14 days post-irradiation). Also, we aimed to possibly identify specific bacterial populations capable of influencing the levels of specific neurotransmitters or precursors in the brain, which were still altered 14 days after the 8 Gy irradiation in comparison to sham control animals. Moreover, as a secondary aim, we explored the possible correlations between post-radiation shifts in neurotransmitter-producing bacteria and significant proteomic changes in the frontal cortex (FCtx), a region of the brain typically involved in the planning, problem-solving, decision-making, motor control, emotions management and social behavior across most large animal species [8,9]. We hypothesize that the FCtx proteomic profiles of animals exposed to lower hemibody irradiation will diverge from the profiles of control animals, suggesting an impact of irradiation on GBA communication.

## 2. Results

### 2.1. Microbiomic Changes Following Gut Irradiation

Quality control analysis of 16S sequencing generated a total frequency of 2,646,000 features retained with a mean frequency of 297,445 per sample (minimum 42.007 and max 1,008,070) after quality control. Sampling depth was set to 42,000. The data has been deposited to NCBI with BioProject ID: PRJNA1274013 at http://www.ncbi.nlm.nih.gov/bioproject/1274013 (accessed on 21 May 2025).

Alpha diversity was not significantly altered by 8 Gy irradiation on day 14, as assessed by Shannon diversity and Faith’s PD (Figure S2A). Beta diversity showed significant non-phylogenic community shifts as evaluated with Bray–Curtis (BC), however non-significant ones when using Generalized UniFrac (GU, phylogenetic) (Figure S2B).

**Figure 1:**
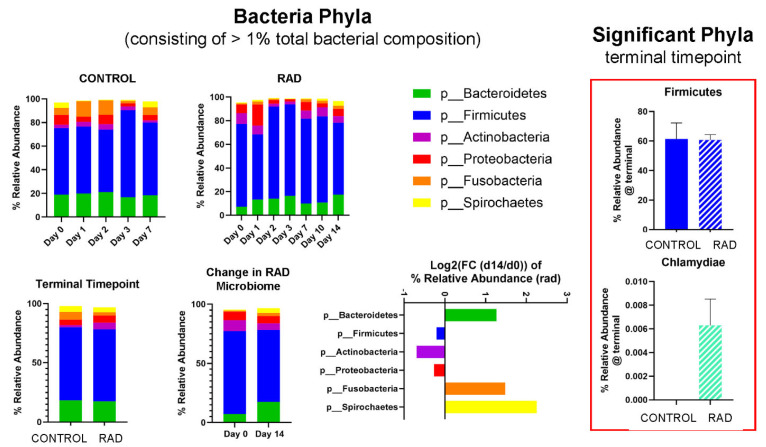
Effects of 8 Gy gut irradiation on gut microbiota at the Phyla level taxonomic classification. Phyla consisting of >1% total bacterial composition are shown for 8 Gy, with mean relative abundance changing over time for CONTROL and RAD groups. Log_2_ (FC) (FC = fold change) of relative abundance for RAD microbiota at d14 compared to baseline is shown. Firmicutes and Chlamydiae were both significantly altered by RAD at d14 vs. CONTROL.

**Figure 2:**
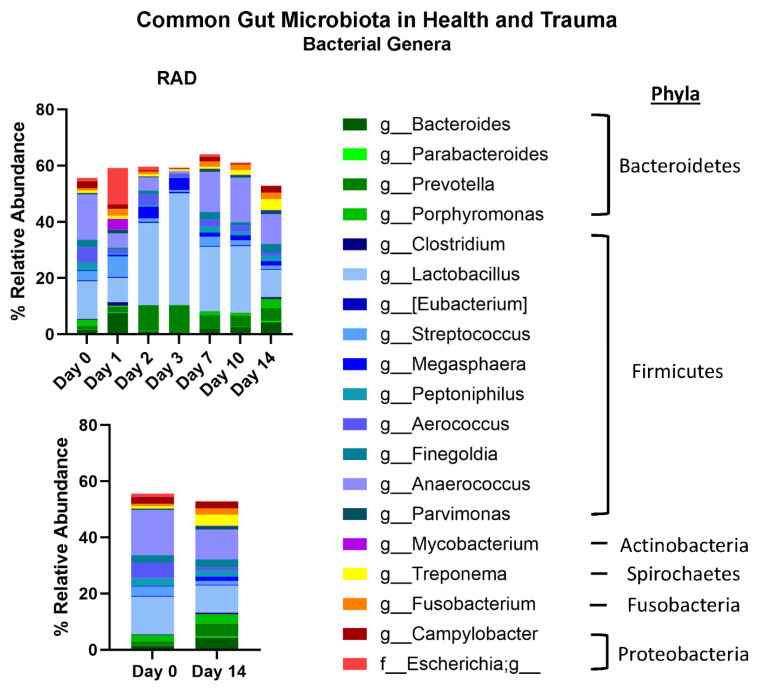
Effects of 8 Gy gut irradiation on common gut microbiome Genera. A panel of common gut microbiota shows how genera change over time after 8 Gy gut irradiation. Genera are color coded to match major Phyla colors in Figure 2.

**Figure 3:**
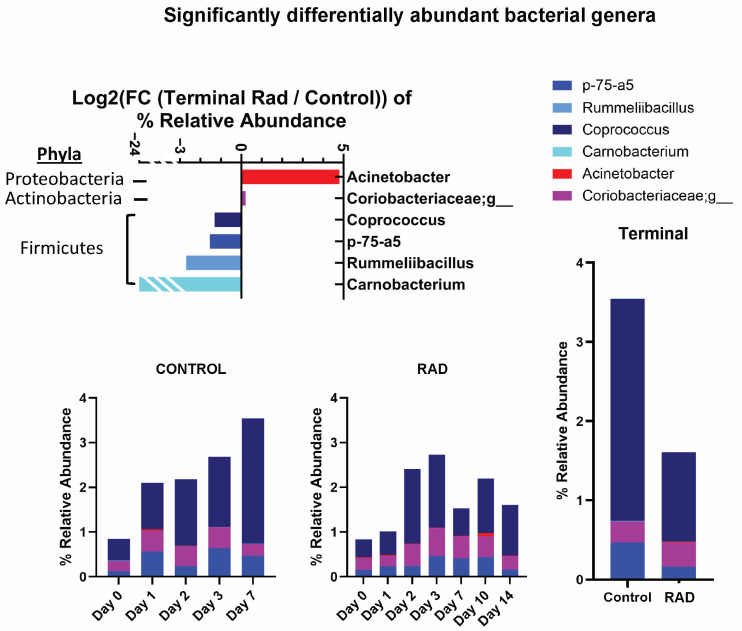
Significantly altered Genera identified following 8 Gy gut irradiation. This level of irradiation primarily affected microbiota of low relative abundance at d14 compared to CONTROL swine. Change in relative abundance was tracked over time. Log_2_ (FC) is presented.

Taxonomic classifications revealed subtle changes in gut microbiota at the phyla and genera level (Table 1). The changes in phyla (consisting of >1% total bacterial composition) are shown in Figure 1, demonstrating how the gut microbiome changes over the course of 14 days post irradiation. At the genera level, we show longitudinal changes of common gut microbiota in health (*Lactobacillus*) and trauma (*Escherichia*) following irradiation (Figure 2). At the terminal timepoint, we saw that radiation decreased the relative abundance of phyla Chlamydiae and Firmicutes (Figure 1), and decreased genera *p-75-a5*, *Coprococcus*, *Rummeliibacillus* and *Carnobacterium*, as well as increased abundance of *Acinetobacter* and an unknown genus of family *Coriobacteriaceae* (Figure 3).

### 2.2. MS Proteomic Profiling

Protein abundance across all samples for proteomic profiling showed optimum quality and consistency for reliable MS-based proteomic analysis (Figure S3A). The unsupervised Principal Component Analysis (PCA) (PC 1: 66.8%; PC 2: 7.9%) graph (Figure S3B) demonstrates some overlap between the RAD (*n* = 6) and Sham/CONTROL (*n* = 6) FCtx group, with several RAD samples approaching the central cluster of the CONTROL group; the heat map (Figure S3C) confirmed this modest separation.

**Figure 4:**
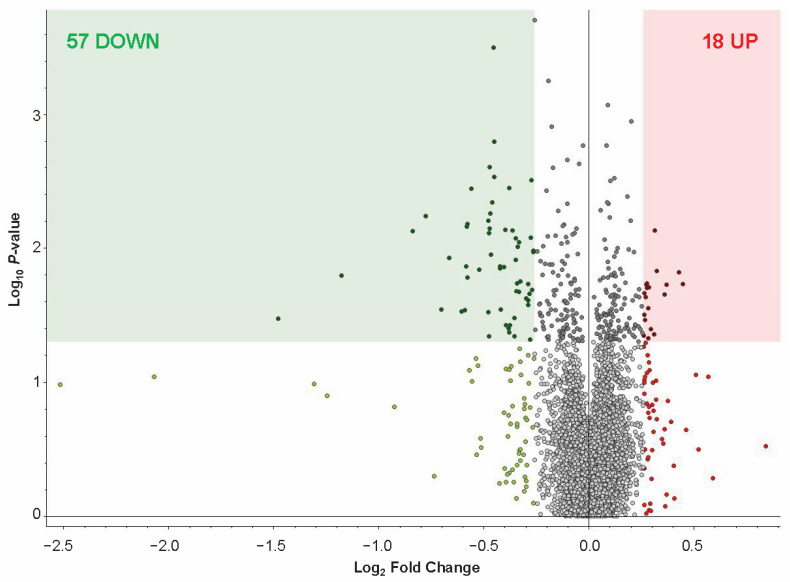
FCtx proteomic profiling of RAD vs. CONTROL swine. (A) Volcano plot of log_2_ (FC) vs. −log10 (*p*-value) of FCtx proteomic profiles, with up-regulated proteins on the right (red) and down-regulated proteins on the left (green). Significance threshold of *p* < 0.05 is indicated by shading, with all significantly changed proteins with log_2_ (FC) ≥ 0.26 included in our analysis shown in the corresponding color shaded boxes (*p*-value of per group ratio calculated by *t*-test; fold changes visualized as log_2_ of abundance ratio). We identified 18 up-regulated (red square) and 57 down-regulated (green square) proteins within these criteria through the proteomic profiling.

MS-proteomic analysis identified 8709 protein groups, with 7186 quantified, from 103,772 peptide groups sequenced from 220,589 peptide-spectral matches at 5% FDR. (see full list of identified proteins in Supplementary Data S1). Based on our thresholding parameters (adjusted *p* < 0.05; Log_2_ (Fold Change (FC)) = 0.26), a total of 18 proteins were increased and 57 proteins were decreased in abundance in the FCtx of RAD vs. CONTROL swine (see list for up- and down-regulated proteins in Supplementary Data S1). A volcano plot of the total increased (red square) and decreased (green square) proteins within our threshold parameters in RAD vs. CONTROL FCtx samples is shown in Figure 4. The TMT-MS raw data and analysis files have been uploaded to the publicly accessible repository MassIVE: MassIVE ID MSV000098819.

### 2.3. STRING Analysis

**Figure 5:**
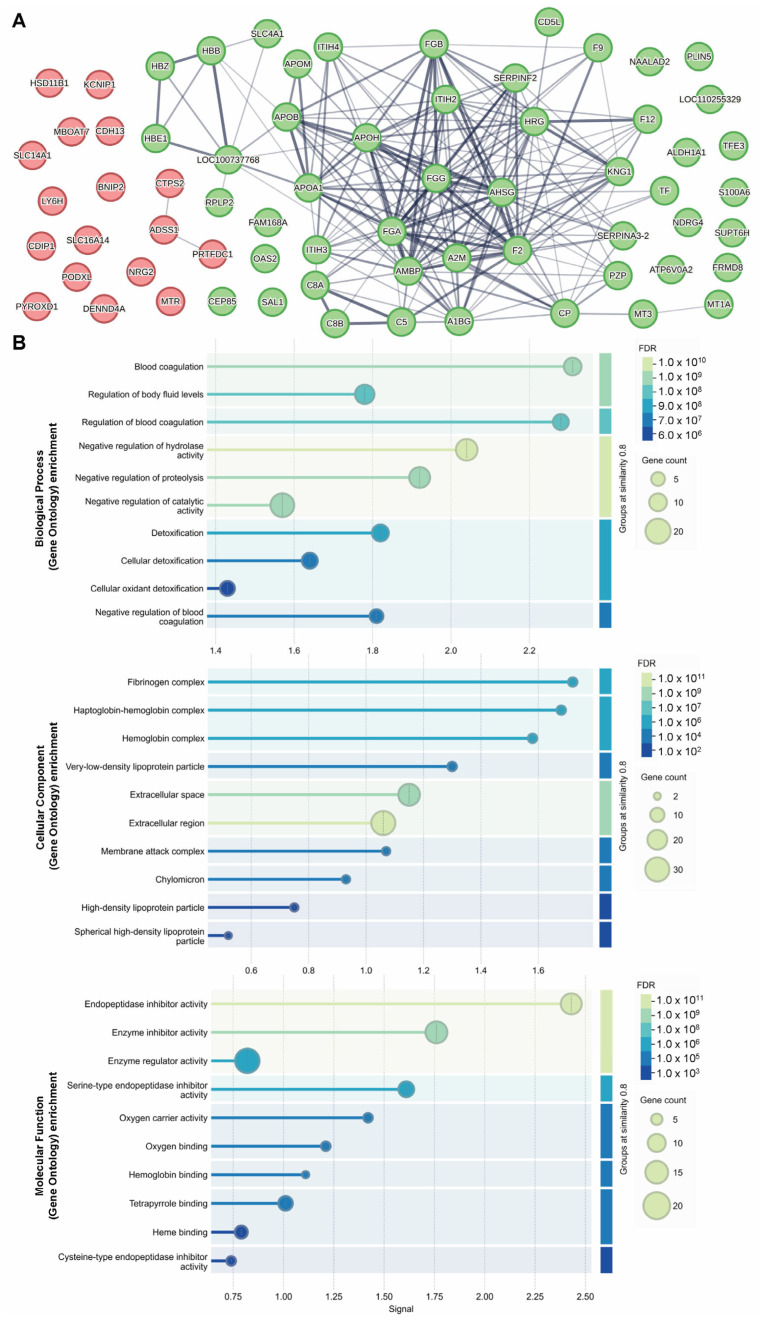
Identifying a network of interactions among up- and down-regulated proteins identified as differentially abundant by MS proteomic analysis. STRING interaction network of proteins with increased (red) and decreased (green) abundance following 8 Gy gut irradiation (**A**). STRING GO analysis results are presented for implicated molecular functions, cellular components and biological processes (**B**).

The STRING database (https://string-db.org/) (accessed on 21 May 2025) is a powerful tool that uses data-mining to provide information regarding protein–protein interactions (known physical interactions and functional associations based on scientific literature, computational predictions from co-expression, genomic content, and experimental data), as well as offering network visualization of the strength of those interactions and functional enrichment analyses [10]. To explore potential associated protein interactions within our up- and down-regulated proteomic targets (72 proteins total input, with 66 identified against Sus scrofa in the STRING database), we used STRING to populate a network (Figure 5A), confirming that these protein interactors are predominantly involved in the inhibition and regulation of enzymatic activity (ITIH2, ITIH3, ITIH4), fibrinogen (FGG, FGB, FGA) and hemoglobin complexes (HBZ, HBE1, HBB), and blood coagulation (HRG, APOH, F2, F9, F12, SLC4A1) (Figure 5B).

### 2.4. DAVID Analysis

The Database for Annotation, Visualization and Integrated Discovery (DAVID) is another powerful tool used to understand biological meaning behind large gene datasets through functional annotation, built on a vast knowledgebase derived from multiple sources (https://david.ncifcrf.gov/) (accessed 20 March 2025) [11,12]. After the list of 71 differentially abundant proteins was input to DAVID for Gene Ontology (GO) analyses against a *Sus scrofa* background, 70 were identified with official gene symbols. Using DAVID’s functional annotation tool, we selected the GO analyses for molecular function (88.6% coverage, includes 62/70 terms from our list), cellular component (97.1% coverage, 68/70 terms) and biological process (92.9% coverage, 65/70 terms). This analysis identified 19 significantly (*p* < 0.05) enriched molecular functions, 19 significantly enriched cellular components, and 41 significantly enriched biological processes (Table 2). The GO results indicate a possible impact of gut irradiation on endopeptidase inhibitor activity, oxygen carrier activity, and oxygen, heme and phospholipid binding (molecular functions), extracellular components, fibrinogen complex and hemoglobin complex (cellular components), as well as fibrinolysis, blood coagulation, negative regulation of endopeptidase activity, platelet activation, complement activation and innate immune response (biological processes) (see Supplementary Data S2).

## 3. Discussion

Previous work from our research group has demonstrated that targeted exposure of the gastrointestinal (GI) tract to high-dose ionizing radiation (8 Gy) induces measurable and lasting alterations in the intestinal microenvironment. In particular, the study by Horseman et al. (2024) [7] identified notable perturbations in the gut microbiota, sustained gut NLRP3 activation, and evidence of impaired intestinal barrier function in these animals 14 days after irradiation. These findings point to a broader systemic effect of localized radiation injury—suggesting that the damage incurred in the gut may have downstream consequences for other organ systems, including the brain. Building on these observations, the current study sought to investigate whether and how gut-targeted radiation modulates brain protein expression, specifically in the frontal cortex (FCtx), a region critical for executive function, cognition, and interoceptive processing.

One of the unique strengths of our experimental approach lies in the use of sexually mature Sinclair minipigs, a large animal model that offers superior anatomical and physiological similarity to humans compared to traditional rodent models. This similarity includes comparable GI tract morphology and microbial communities, as well as brain organization and size, making the minipig a particularly relevant model for studying the gut–brain axis (GBA). Moreover, the use of localized lower hemibody irradiation ensured that the brain was anatomically shielded from direct radiation exposure, allowing us to assess true abscopal effects—systemic biological responses to localized treatment—in the CNS. To our knowledge, this study represents the first investigation of the effects of high-dose ionizing radiation targeted to the gut on both the gut microbiota and the brain proteome in a large mammalian model. Importantly, we focused on a highly specialized brain region, the frontal cortex, rather than assessing global brain changes. The primary objective was to determine whether proteomic alterations in the FCtx occur following gut irradiation and to explore whether these changes might be mediated, at least in part, by radiation-induced shifts in the intestinal microbiota.

While the full gut microbiome dataset has been previously published [7], our current analysis was restricted to swine exposed to the 8 Gy dose group, excluding the 10 and 12 Gy cohorts. The rationale for this selection was twofold. First, the 8 Gy dose produced robust gut effects without incurring mortality within the 14-day observation window. Second, animals in the 8 Gy group showed resolution of clinical signs of distress (e.g., lethargy, diarrhea, anorexia) by day 14, minimizing potential confounds related to acute systemic illness or terminal decline. We therefore deemed this group optimal for assessing microbiota-related effects on the brain that are not clouded by severe systemic compromise. Rectal swabs collected longitudinally were reanalyzed to focus on taxa significantly altered between irradiated and control animals at day 14, the same timepoint used for frontal cortex tissue collection and proteomic profiling.

The beta diversity metrics shown in Figure 1 reveal that gut microbiota community composition was modestly significantly different between RAD and CONTROL at termination, not accounting for evolutionary relationships between taxa. Looking at the longitudinal changes of common gut bacterial genera (Figure 2), we see an acute decrease of beneficial flora and an increase in Escherichia (opportunistic pathogen) d1 post-irradiation, followed by a period of almost a compensatory response of beneficial flora before returning close to baseline by d10–14. This suggests that much of the impact of radiation on the gut microbiome occurs during the first few days post exposure. At the phyla level, although the relative abundance of Firmicutes appeared only modestly elevated in the rad group at d14 (60.7% vs. 61.4%) (Figure 1), the difference in differential abundance may reflect a consistent compositional shift detectable by Analysis of Compositions of Microbiomes with Bias Correction (ANCOM-BC) rather than a large effect size. Given previous findings of increased Firmicutes abundance at earlier timepoints (e.g., Day 2), these results may suggest a biphasic or temporally dynamic response to radiation. It is also possible that late-phase changes in community structure within Firmicutes contribute to this, despite relatively stable phylum-level totals.

MS-based proteomic analysis of the FCtx revealed moderate but significant changes in protein abundance following gut irradiation. Specifically, we identified 57 proteins with decreased abundance and 18 proteins with increased abundance (*p* < 0.05, fold change ≥ 2) in irradiated versus sham control animals. These high-confidence proteins were subsequently analyzed using STRING and DAVID platforms for functional network and gene ontology enrichment. The results indicated that the predominant proteomic changes in the FCtx involved pathways related to immune signaling, oxygen transport, fibrinogen, and hemoglobin activity. Notably, these findings suggest that the brain may initiate a compensatory or adaptive response to distal tissue injury in the gut, potentially through immune or vascular signaling pathways, despite being physically shielded from direct radiation exposure.

Our findings also provide novel insight into the possible role of the inflammasome in mediating gut–brain crosstalk following radiation injury. Horseman et al. (2024) [7] reported a marked upregulation of NLRP3 and TLR4 in jejunal lysates of irradiated swine, implicating the NLRP3 inflammasome in the gut’s inflammatory response to radiation. Consistent with this, our FCtx proteomic data showed a significant decrease in pannexin-1 (PANX1; *p* = 0.03, log_2_ (FC) = −0.23). PANX1 is a membrane channel protein known to facilitate ATP release and acts as an upstream regulator and activator of the NLRP3 inflammasome [13,14]. The downregulation of PANX1 in the brain suggests that systemic inflammatory feedback following gut irradiation may suppress inflammasome-related activity in the brain, representing a potential homeostatic or neuroprotective mechanism. This molecular crosstalk could be mediated by several neuroimmune mechanisms. For instance, gut damage can trigger the release of pro-inflammatory cytokines (e.g., TNF-α, IL-6, IL-1β) into circulation, which can cross the blood–brain barrier and influence CNS immune cells [15]. Alternatively, signals may be transmitted via the vagus nerve, which directly connects the gut to the brainstem [2]. Furthermore, microbial metabolites, such as short-chain fatty acids (SCFAs), are known to modulate microglial activation and neuroinflammation, and their production is highly dependent on the composition of the gut microbiota [16]. These data strengthen the hypothesis that the GBA mediates molecular crosstalk between the gut and the brain in the context of localized radiation injury.

We next explored how changes in the microbiota may underlie or contribute to the observed FCtx proteomic alterations. A growing body of literature has demonstrated that specific gut bacteria can influence host neurotransmitter production and signaling, either through direct synthesis of neurotransmitters (e.g., GABA, serotonin) or via modulation of host precursors and enzymes [17,18]. To this end, we focused our analysis on bacterial taxa associated with known neurotransmitter pathways and searched for overlapping proteomic changes in the FCtx related to these signaling systems (Table 3).

While we present these observed correlations as exploratory, it is important to note that the individual genera linked to neurotransmitter activity did not show statistically significant changes in abundance in our 8 Gy microbiome dataset. This weakens any direct causal inference. However, we note that several of these genera are nested within the Firmicutes phylum, which was significantly reduced in irradiated animals. This suggests a potential speculative link where a radiation-induced disruption of Firmicutes may impair neurotransmitter signaling indirectly.

Although individual genera linked to neurotransmitter activity did not show statistically significant changes in abundance in our 8 Gy microbiome dataset, several of these genera are nested within the Firmicutes phylum, which was significantly reduced in irradiated animals. This suggests that radiation-induced disruption of Firmicutes may impair neurotransmitter signaling indirectly. To investigate further, we cross-referenced the altered phyla and genera with published associations to neuronal signaling (Table 4) and queried our FCtx proteomic dataset for corresponding proteins involved in neurotransmission (Table 5). Notable candidates included GABRA3 (GABA receptor subunit alpha-3), PTPRG (a tyrosine phosphatase linked to synaptic regulation), CHRM1 (muscarinic acetylcholine receptor M1), and TPH2 (tryptophan hydroxylase 2, the rate-limiting enzyme in serotonin biosynthesis). These proteins were altered in irradiated animals, albeit at varying statistical thresholds, warranting further targeted validation.

While causality cannot be firmly established, our integrated analysis supports a potential influence of gut microbiota dysbiosis on brain neurotransmitter-related signaling. Importantly, we note that the most dramatic shifts in microbiota composition occurred early in the post-irradiation timeline, suggesting that transient microbial disruption may trigger downstream signaling cascades that culminate in proteomic changes in the brain days later. Longitudinal studies with more frequent timepoint sampling of the brain are needed to fully resolve the temporal dynamics of this gut–brain communication. We know from comparing beta diversity PCAs in Horseman et al. (2024) [7] that perturbations to the gut microbiome communities largely resolve by day 14 after 8 Gy irradiation, though higher doses demonstrate a lingering effect; how these modulations of the gut microbiota influence communication along the GBA over time remains to be elucidated.

Our findings may have important clinical implications. Patients receiving pelvic radiation for cancer often suffer from long-term neurological symptoms, including cognitive impairment and mood disorders, which are poorly understood [32]. Our results suggest that radiation-induced gut dysbiosis could be a contributing factor. This opens the door to exploring microbiota-targeted interventions, such as tailored probiotics, prebiotics, or even fecal microbiota transplantation (FMT), as potential strategies to mitigate the adverse neuroimmune effects of radiation therapy and improve patients’ quality of life.

In future investigations, we plan to use immunohistochemistry and Western blotting to validate the brain proteins of interest with the greatest fold changes. In addition to the FCtx, we aim to assess these proteins in other brain regions implicated in GBA communication, such as the hippocampus, amygdala, and brainstem, as well as in the vagus nerve—a major anatomical conduit for gut–brain signaling. These complementary methods will help determine whether the observed proteomic changes are region-specific or reflect a broader neurobiological response to gut injury.

An important consideration emerging from our study is the potential relevance of gut irradiation to neurodegenerative processes. Recent clinical and preclinical studies have linked gut dysbiosis to the pathogenesis of neurodegenerative and psychiatric disorders, including Alzheimer’s disease, Parkinson’s disease, and depression [33,34,35]. Given this, we screened our FCtx proteomic dataset for established markers of neuroinflammation and neurodegeneration but did not detect significant changes at the 14-day timepoint (Table 6). This may be due to the relatively short post-irradiation interval; longer-term studies allowing animals to age past acute and subacute phases may be necessary to detect chronic or progressive neurodegenerative changes.

Several limitations of our study must be acknowledged. First, the sample size was relatively small, which may limit the generalizability of our findings and reduce statistical power for detecting subtle effects. Second, only male animals were included; sex-specific differences in microbiota composition and GBA signaling have been documented and merit future investigation. Third, the control group was euthanized at day 7, while irradiated animals were analyzed at day 14, creating a potential temporal confound, although no significant health differences were observed by day 14. Fourth, other systemic factors, such as the physiological stress response to the irradiation procedure itself, were not measured. Stress hormones like corticosterone are known to impact both the gut microbiome and brain function, representing a potential confounding variable that should be assessed in future work. Fifth, the use of antibiotics for post-irradiation care is a significant confounder. Antibiotics themselves are potent modulators of the gut microbiota and can influence the gut–brain axis independently of radiation exposure. The observed changes could therefore be a result of the radiation, the antibiotics, or a synergistic interaction between the two. Future studies are critically needed and should include an antibiotic-only control group to disentangle these effects.

Despite these limitations, our findings underscore the utility of large animal models for studying the systemic effects of localized radiation and illuminate novel aspects of gut–brain communication. Building on this work, future investigations should aim to validate the key protein changes with methods like immunohistochemistry and Western blotting, not only in the FCtx but also in other key GBA-related brain regions like the hippocampus, amygdala, and brainstem. To definitively establish a causal role for the microbiota, studies involving the transplantation of fecal microbiota from irradiated donors into germ-free or antibiotic-treated recipients would be invaluable. Finally, measuring systemic and central inflammatory markers, such as plasma and brain cytokines (e.g., IL-1β, IL-18), will be essential to directly test the neuroinflammatory hypotheses generated by this study. These approaches will help determine whether the observed proteomic changes are region-specific or reflect a broader neurobiological response to gut injury, with significant implications for understanding the microbiota-neuroimmune interface in health and disease.

## 4. Material and Methods

The detailed experimental setup, including radiation procedures, timing, dose rate, and observed hematological and physical symptoms following different doses of GI-irradiation, including 8 Gy irradiation, has been described previously [7]. Succinctly, adult male Sinclair minipigs were assigned to either the radiation group (RAD, *n* = 6), receiving a single 8 Gy dose, or the control group (CONTROL, *n* = 6), which underwent only catheter implantation. Radiation was administered as a lower hemi-body exposure using a clinical linear accelerator (Elekta Infinity, Armed Forces Radiobiology Research Institute LINAC) delivering 4 MV photons at a dose rate of 1.9 Gy/min. A summary of animal features is presented in Figure S1. Animal health, including clinical signs and weight, was monitored throughout the study.

All research was approved by the Institutional Animal Care and Use Committee (IACUC) at the Uniformed Services University of the Health Sciences and conducted in an AAALAC-accredited facility, following the Guide for the Care and Use of Laboratory Animals and the Animal Welfare Act. Here, we outline the key experimental methods and procedures used to assess proteomic changes in the brains of irradiated swine compared to sham controls.

### 4.1. Tissue Sample Preparation

As previously described in detail [7], animals were euthanized via jugular catheter administration of Euthasol (100 mg/kg, Virbac, Carros, France) after 7 days (Sham/Control, [SH]) or 14 days of recovery (RAD). Brains were then extracted from the skull. The left hemisphere was flash-frozen in chilled liquid isopentane on dry ice for molecular analyses and stored at −80 °C until use.

Frozen left hemispheres were sectioned on a cryostat at −20 °C into 100 µm thick coronal sections and microdissected to isolate the frontal cortex (FCtx) and other regions (not discussed here), following atlas-guided anatomical delineations.

For proteomic analysis, FCtx tissue (~600–900 mg per animal) was homogenized as described previously [36]. Total protein content was quantified using the Micro BCA assay (Thermo Fisher Scientific, 23235, Waltham, MA, USA). Briefly, FCtx tissue from each biological replicate was homogenized in ice-cold lysis buffer using glass dounce homogenizers, centrifuged, and the supernatant collected, aliquoted, and frozen. Samples were subsequently used for MS-based proteomic analysis, with potential additional Western blot validation.

### 4.2. 16S rRNA Sequencing-Based Microbiome Analysis Procedures

DNA was isolated from rectal swabs using the QIAmp PowerFecal Pro kit (Qiagen, Germantown, MD, USA) without modifications. Library preparation was performed using the “Illumina 16S Metagenomic Sequencing Library Preparation” protocol with 16S V4 region 515F–806R primers [37,38]. DNA and libraries were quantified using Qubit 4.0 (Thermo Fisher Scientific, Waltham, MA, USA). Sequencing was performed on a NextSeq 500 using a Mid Output v2.5 2 × 150 bp kit (Illumina, San Diego, CA, USA). Bioinformatic analysis was performed through previously described pipelines [39], with DADA2 for feature table generation based on a median quality score of >25 [40,41,42], which excluded reverse reads. The resulting table were imported into Quantitative Insights Into Microbial Ecology (QIIME2, version 2023.7) [43], with taxonomy analyzed against the Greengenes database. Further details and tools on the analysis pipeline were informed from the literature and can be found in Horseman et al. (2024) [7].

### 4.3. Tandem Mass Tag (TMT) Proteomics Procedures and Data Analysis

*Isobaric Mass Tag Labeling and Fractionation:* Protein extracts were buffer exchanged using SP3 paramagnetic beads (GE Healthcare, Chicago, IL, USA) [44]. Briefly, samples were brought up to 100 µL with 10 mM TEAB + 1% SDS and disulfide bonds reduced with 10 µL of 50 mM dithiothreitol for 1 h at 60 °C. Samples were cooled to RT and pH adjusted to ~7.5, followed by alkylation with 10 µL of 100 mM iodoacetamide in the dark at RT for 15 min. Next, 100 ug (2 µL of 50 ug/µL) SP3 beads were added to the samples, followed by 120 µL 100% ethanol. Samples were incubated at RT with shaking for 5 min. Following protein binding, beads were washed with 180 µL 80% ethanol three times. Proteins were digested on-bead with trypsin (Pierce) (Thermo Fisher Scientific, Waltham, MA, USA) at 37 °C overnight (5 ug enzyme) and labeled with a unique TMTpro 16-plex reagent (Thermo Fisher Scientific, LOT # YK388744, Waltham, MA, USA) according to the manufacturer’s instructions. All 12 TMT labeled peptide samples were combined, dried by vacuum centrifugation, resuspended in 100 µL 200 mM TEAB buffer and filtered through Pierce Detergent removal columns (Thermo Fisher Scientific, PN 87777, Waltham, MA, USA) to remove excess TMT label, small molecules and lipids. Peptides in the flow through were diluted to 2 mL in 10 mM TEAB and fractionation on a XBridge C18 Column (5 µm, 2.1 × 100 mm column (Waters) using a 0 to 90% acetonitrile in 10 mM TEAB gradient over 85 min at 250 µL/min on an Agilent 1200 series capillary HPLC with a micro-fraction collector. Eighty-four 250 µL fractions were collected and concatenated into 24 fractions according to Wang et al. (2011) [45] and dried by vacuum centrifugation.

*Mass Spectrometry analysis:* Peptides in each of the 24 fractions were analyzed by reverse-phase chromatography tandem mass spectrometry on a Neo Vanquish UPLC interfaced with an Orbitrap Exploris 480 mass spectrometer (Thermo Fisher Scientific, Waltham, MA, USA). Peptides were introduced onto a 2 cm trapping column before being separated using a 2–95% acetonitrile in 0.1% formic acid gradient over 90 min at 300 nL/min. The 75 µm × 25 cm column was packed in house with ReproSIL-Pur-120-C18-AQ (2.4 µm, 120 Å bulk phase, Dr. Maisch) and ionized at 2200 volts using a Pepsep Emitter setup (Bruker, Billerica, MA, USA). MS2 from survey scans of precursor ions were acquired in a 3 s cycle time, from 375–1500 m/z at 120,000 resolution at 200 m/z, automatic gain control (AGC) of 3 × 10^6^. TheRF lens was set to 45% and the internal mass calibration of the instrument was enabled. Precursor ions were individually isolated within 0.7 m/z by data dependent acquisition with a 45s dynamic exclusion, and fragmented using an HCD activation with a collision energy of 36. Fragment ions were analyzed at 30,000 resolution and an AGC setting of 1 × 10^5^ with TurboTMTPro setting enabled.

*Data analysis:* Fragmentation spectra were processed by Proteome Discoverer v3.1 (PD3.1 ThermoFisher Scientific, Waltham, MA, USA) and searched with CHIMERYS using inferys 3.0.0 fragmentation model against the Uniprot Sus scrofa database downloaded on 240729. Search criteria included trypsin as the enzyme, two missed cleavages, and 20 ppm tolerance on fragment ions. TMTpro on N-terminus and K and carbamidomethylation on C were set as fixed modifications andoxidation on M was set to variable. Peptide identifications from the CHYMERYS searches were processed within PD3.1 using Percolator at a 1% False Discovery Rate. Peptide spectral matches (PSMs) were filtered for Isolation Interference with a Normalized CHIMERYS Coefficient of 0.8. Relative protein abundances of identified proteins were determined in PD3.1 from the normalized median ratio of TMT reporter ions, having signal to noise ratios > 4, from all PSMs from the same protein. Technical variation in ratios from our mass spectrometry analysis is less than 10% [46].

### 4.4. Statistics

For microbiomic data, statistical testing for alpha diversity was conducted with a two-way ANOVA with Tukey’s or Dunnett’s post-testing. A permutational analysis of variance (PERMANOVA) was used to assess pairwise associations in beta diversity measures. Unless otherwise stated, values are represented as mean ± SD.

For the MS-proteomics data analyses and methods see section “Tandem Mass Tag (TMT) proteomics procedures and data analysis”. *p*-values were calculated using *t*-test for individual proteins with biological replicates. To account for multiple comparisons, a Benjamini–Hochberg false discovery rate (FDR) correction was applied, with an adjusted *p*-value (q-value) < 0.05 considered significant. Grouped abundance coefficient of variation (CV) = 100 × std. dev/median. Z-score transformation of normalized protein abundances from a quantitative proteomics analysis using isobaric mass tags was applied before performing the hierarchical clustering based on Euclidean distance and complete (furthest neighbors) linkage.

Statistical analysis and figure generation were performed using Prism 10.4.2 (GraphPad Software, San Diego, CA, USA) unless otherwise noted. Statistical significance was set as *p* < 0.05.

## Figures and Tables

**Table 1:** Altered gut microbiome 14 days post 8 Gy gut irradiation. Two phyla and six genera were identified to be significantly changed at the terminal timepoint comparing RAD and CONTROL swine microbiomes.

	Bacteria ID Significant at Terminal Timepoint	Adj *p* Value	Direction of Change
**Phyla**	k__Bacteria; p__**Chlamydiae**	9.8 × 10^−4^	Down
	k__Bacteria; p__**Firmicutes**	9.9 × 10^−4^	Down
**Genera**	k__Bacteria; p__Firmicutes; c__Erysipelotrichi; o__Erysipelotrichales; f__Erysipelotrichaceae; g__**p-75-a5**	5.98 × 10^−15^	Down
	k__Bacteria; p__Actinobacteria; c__Coriobacteriia; o__Coriobacteriales; f__**Coriobacteriaceae;g__**	9.6 × 10^−4^	Up
	k__Bacteria; p__Firmicutes; c__Bacilli; o__Bacillales; f__Planococcaceae; g__**Rummeliibacillus**	0.003	Down
	k__Bacteria; p__Proteobacteria; c__Gammaproteobacteria; o__Pseudomonadales; f__Moraxellaceae; g__**Acinetobacter**	0.003	Up
	k__Bacteria; p__Firmicutes; c__Clostridia; o__Clostridiales; f__Lachnospiraceae; g__**Coprococcus**	0.009	Down
	k__Bacteria; p__Firmicutes; c__Bacilli; o__Lactobacillales; f__Carnobacteriaceae; g__**Carnobacterium**	0.014	Down

**Table 2:** DAVID analyses of differentially abundant FCtx proteins identified through proteomic analysis of RAD vs. CONTROL swine. Following input of 71 differentially abundant proteins identified through proteomic analysis of the FCtx after gut irradiation, DAVID enrichment analysis of GO relationships revealed 19 molecular functions, 19 cellular components and 41 biological processes as potentially significantly affected.

	Term	Count	%	*p*-Value	Genes
**Molecular Function**	serine-type endopeptidase inhibitor activity	10	14.28571	1.64 × 10^−10^	*LOC396685, ITIH4, ITIH3, ITIH2, AMBP, PZP, SERPINF2, HRG, A2M, LOC100153899*
	endopeptidase inhibitor activity	4	5.714286	3.16 × 10^−5^	*C5, AHSG, PZP, A2M*
	signaling receptor binding	7	10	6.64 × 10^−5^	*FGB, FGA, FGG, NRG2, F2, HRG, A2M*
	haptoglobin binding	3	4.285714	3.77 × 10^−4^	*HBZ, HBE1, HBB*
	organic acid binding	3	4.285714	5.74 × 10^−4^	*HBZ, HBE1, HBB*
	oxygen carrier activity	3	4.285714	0.0010859	*HBZ, HBE1, HBB*
	oxygen binding	3	4.285714	0.0021445	*HBZ, HBE1, HBB*
	heme binding	5	7.142857	0.0029643	*HBZ, AMBP, HBE1, HBB, HRG*
	phospholipid binding	4	5.714286	0.0031406	*APOM, APOH, APOA1, APOB*
	cysteine-type endopeptidase inhibitor activity	3	4.285714	0.0043586	*AHSG, HRG, KNG1*
	peroxidase activity	3	4.285714	0.0049488	*HBZ, HBE1, HBB*
	heparin binding	4	5.714286	0.0101698	*APOH, F2, APOB, HRG*
	oxidoreductase activity	4	5.714286	0.0178829	*AMBP, PYROXD1, ALDH1A1, CP*
	hemoglobin alpha binding	2	2.857143	0.0195663	*HBE1, HBB*
	calcium ion binding	7	10	0.0241982	*F9, KCNIP1, F12, S100A6, CDH13, F2, MBL1*
	serine-type endopeptidase activity	4	5.714286	0.0260039	*F9, F12, CD5L, F2*
	protease binding	3	4.285714	0.0346761	*PZP, SERPINF2, A2M*
	protein homodimerization activity	6	8.571429	0.0416447	*HSD11B1, AMBP, SERPINF2, S100A6, APOA1, SLC4A1*
	immunoglobulin receptor binding	2	2.857143	0.0419182	*IGHM, LOC100125542*
**Cellular Component**	extracellular space	27	38.57143	1.16 × 10^−13^	*CD5L, PZP, INHCA, C8B, C8A, KNG1, LOC396685, C5, SAL1, APOH, APOB, A2M, MBL1, LOC100153899, FGB, AHSG, F12, FGG, SERPINF2, APOA1, NRG2, F2, CP, TF, F9, S100A6, CDH13*
	extracellular region	25	35.71429	1.24 × 10^−13^	*ITIH4, ITIH3, ITIH2, PZP, INHCA, A1BG, C8B, C8A, KNG1, C5, APOH, A2M, MBL1, FGB, FGA, AMBP, AHSG, F12, APOA1, NRG2, F2, CP, TF, F9, HRG*
	blood microparticle	7	10	1.69 × 10^−9^	*HBZ, AHSG, HBE1, HBB, LOC100125542, HRG, KNG1*
	fibrinogen complex	4	5.714286	6.52 × 10^−7^	*FGB, FGA, FGG, SERPINF2*
	very-low-density lipoprotein particle	4	5.714286	1.16 × 10^−5^	*APOM, APOH, APOA1, APOB*
	membrane attack complex	3	4.285714	2.16 × 10^−4^	*C5, C8B, C8A*
	low-density lipoprotein particle	3	4.285714	2.88 × 10^−4^	*APOM, APOA1, APOB*
	haptoglobin-hemoglobin complex	3	4.285714	5.62 × 10^−4^	*HBZ, HBE1, HBB*
	hemoglobin complex	3	4.285714	5.62 × 10^−4^	*HBZ, HBE1, HBB*
	chylomicron	3	4.285714	6.73 × 10^−4^	*APOH, APOA1, APOB*
	high-density lipoprotein particle	3	4.285714	0.0013715	*APOM, APOH, APOA1*
	cell surface	6	8.571429	0.0120099	*IGHM, TF, AMBP, APOH, SERPINF2, HRG*
	collagen-containing extracellular matrix	4	5.714286	0.0163355	*FGB, FGG, S100A6, F2*
	spherical high-density lipoprotein particle	2	2.857143	0.0225352	*APOM, APOA1*
	cytoplasmic side of plasma membrane	3	4.285714	0.0260414	*KCNIP1, S100A6, SLC4A1*
	chromaffin granule	2	2.857143	0.0351871	*LOC396685, LOC100153899*
	external side of plasma membrane	5	7.142857	0.0393067	*FGB, FGG, CDH13, LOC100125542, F2*
	platelet alpha granule	2	2.857143	0.0414524	*FGB, FGG*
**Biological Process**	fibrinolysis	6	8.571429	1.83 × 10^−10^	*FGB, FGA, F12, FGG, F2, HRG*
	blood coagulation	8	11.42857	3.03 × 10^−10^	*FGB, FGA, F9, F12, FGG, SLC4A1, F2, HRG*
	negative regulation of endopeptidase activity	6	8.571429	6.83 × 10^−7^	*LOC396685, AHSG, SERPINF2, HRG, KNG1, LOC100153899*
	platelet activation	5	7.142857	1.50 × 10^−6^	*FGB, FGA, FGG, F2, HRG*
	complement activation, classical pathway	5	7.142857	3.21 × 10^−6^	*C5, LOC100125542, C8B, C8A, MBL1*
	innate immune response	8	11.42857	1.07 × 10^−4^	*FGB, IGHM, FGA, C5, OAS2, LOC100125542, C8B, C8A*
	negative regulation of fibrinolysis	3	4.285714	1.72 × 10^−4^	*APOH, SERPINF2, HRG*
	positive regulation of ERK1 and ERK2 cascade	6	8.571429	2.91 × 10^−4^	*FGB, FGA, NDRG4, FGG, SERPINF2, MT3*
	positive regulation of peptide hormone secretion	3	4.285714	3.19 × 10^−4^	*FGB, FGA, FGG*
	hyaluronan metabolic process	3	4.285714	4.09 × 10^−4^	*ITIH4, ITIH3, ITIH2*
	plasminogen activation	3	4.285714	4.09 × 10^−4^	*FGB, APOH, FGG*
	positive regulation of heterotypic cell–cell adhesion	3	4.285714	6.23 × 10^−4^	*FGB, FGA, FGG*
	complement activation, alternative pathway	3	4.285714	6.23 × 10^−4^	*C5, C8B, C8A*
	cellular oxidant detoxification	3	4.285714	7.46 × 10^−4^	*HBZ, HBE1, HBB*
	protein polymerization	3	4.285714	8.79 × 10^−4^	*FGB, FGA, FGG*
	oxygen transport	3	4.285714	8.79 × 10^−4^	*HBZ, HBE1, HBB*
	complement activation	3	4.285714	0.001706	*C5, C8B, C8A*
	acute-phase response	3	4.285714	0.0027901	*ITIH4, AHSG, F2*
	negative regulation of cell adhesion	3	4.285714	0.0035605	*PODXL, CDH13, KNG1*
	negative regulation of extrinsic apoptotic signaling pathway via death domain receptors	3	4.285714	0.0035605	*FGB, FGA, FGG*
	zymogen activation	3	4.285714	0.0035605	*F9, F12, CD5L*
	hydrogen peroxide catabolic process	3	4.285714	0.0035605	*HBZ, HBE1, HBB*
	cholesterol efflux	3	4.285714	0.0035605	*APOM, APOA1, APOB*
	platelet aggregation	3	4.285714	0.005696	*FGB, FGA, FGG*
	antibacterial humoral response	3	4.285714	0.0082861	*IGHM, TF, INHCA*
	iron ion transport	3	4.285714	0.0082861	*TF, INHCA, CP*
	positive regulation of phagocytosis	3	4.285714	0.0082861	*AHSG, APOA1, MBL1*
	cytolysis by host of symbiont cells	2	2.857143	0.0102421	*F2, HRG*
	negative regulation of endothelial cell apoptotic process	3	4.285714	0.0132219	*FGB, FGA, FGG*
	negative regulation of blood coagulation	2	2.857143	0.0170127	*APOH, KNG1*
	adaptive immune response	4	5.714286	0.0190219	*FGB, IGHM, FGA, LOC100125542*
	blood coagulation, fibrin clot formation	2	2.857143	0.0203809	*FGB, FGG*
	lipoprotein biosynthetic process	2	2.857143	0.0237378	*APOA1, APOB*
	high-density lipoprotein particle assembly	2	2.857143	0.0270833	*APOM, APOA1*
	detoxification of copper ion	2	2.857143	0.0304175	*MT1A, MT3*
	positive regulation of blood coagulation	2	2.857143	0.0337405	*F12, F2*
	reverse cholesterol transport	2	2.857143	0.0403528	*APOM, APOA1*
	positive regulation of collagen biosynthetic process	2	2.857143	0.0403528	*SERPINF2, F2*
	negative regulation of proteolysis	2	2.857143	0.0436423	*F2, KNG1*
	high-density lipoprotein particle remodeling	2	2.857143	0.0436423	*APOM, APOA1*
	cellular response to zinc ion	2	2.857143	0.0469207	*MT1A, MT3*

**Table 3:** Known associations [18] between gut microbiota and neurotransmitter synthesis in the gut–brain axis. Corresponding direction of change noted for RAD vs. CONTROL samples (none significant). Neural transmitters are color coded to match other tables. Phyla are color coded to match Figure 2.

Neuronal Associations [18]	Genera	Phyla	Direction	Log_2_ (FC)
Glutamate	Lactobacillus	**Firmicutes**	Down	−2.51933
Acetylcholine				
Glutamate	Bacteroides	**Bacteroidetes**	Down	−1.00345
GABA				
Glutamate	Campylobacter	**Proteobacteria**	Down	−0.64412
GABA	Bifidobacterium	**Actinobacteria**	Down	−0.77407
GABA	Parabacteroides	**Bacteroidetes**	Down	−1.67344
GABA	Eubacterium	**Firmicutes**	Up	0.437964
Acetylcholine	Bacillus	**Firmicutes**	Up	0.97433
Acetylcholine	Staphylococcus	**Firmicutes**	Down	−0.77694
Dopamine				
Serotonin				
Tyramine				
Phenylethylamine				
Tryptamine				
Tyramine	Providencia	**Proteobacteria**	Down	−0.23084
Tryptamine	Ruminococcus	**Firmicutes**	Down	−1.38384
Tryptamine	Clostridium	**Firmicute**	Up	0.918807

**Table 4:** Significant changes to gut microbiome at d14 post 8 Gy irradiation. Known neural associations for specific phyla and genera are noted. *p*-values and direction of change are listed under each bacterium. Neurotransmitters are color coded to match other tables.

Neuronal Associations	Phyla	Genera	Neuronal Associations
	**Chlamydiae**		
Serotonin [19]	d14 *p* = 0.00098		
	Down		
		**p-75-a5**	
		d14 *p* = 5.98 × 10^−15^	Glutamate [20]
		Down	
Serotonin [21]		**Rummeliibacillus**	
GABA [22]	**Firmicutes**	d14 *p* = 0.00261	N/A
Dopamine [23]	d14 *p* = 0.00099	Down	
Acetylcholine [18]	Down	**Coprococcus**	SCFA [24]
Norepinephrine		d14 *p* = 0.00854	Serotonin [25]
		Down	
		**Carnobacterium**	
		d14 *p* = 0.01426	Tyrosine [26]
		Down	
Norepinephrine [27]	Proteobacteria	**Acinetobacter**	
GABA [28]	(ns)	d14 *p* = 0.00328	N/A
Serotonin [29]		Up	
Serotonin [30]	Actinobacteria	**f__Coriobacteriaceae;g__**	
GABA	(ns)	d14 *p* = 0.00096	Serotonin [31]
Dopamine		Up	

**Table 5:** Significant proteomic changes in the FCtx associated with neuronal signaling. Neurotransmitters are color coded to match Table 3 and Table 4. Log_2_ (FC) and *p*-values are listed for these significant proteins.

Neuronal Associations	FCtx Protein	Log_2_ (FC)	*p*-Value
GABA	GABBR1	0.05	0.044610083
	GABRA3	0.15	0.048704857
Glutamate	GRM3	−0.19	0.030625303
	GMPS	−0.03	0.032725995
	GRIA1	0.14	0.010037585
Norepinephrine	ACP1	0.05	0.017387287
Dopamine	PTPN9	0.08	0.045813215
	PTPRG	0.17	0.026421571
NAAG	NAALAD2	−0.52	0.014497586
Acetylcholine	CHRM1	0.12	0.023931344
Serotonin	KYAT1	0.06	0.005224157
	TPH2	−0.23	0.02642157
Histamine	HRG	−0.42	0.013733175

**Table 6:** Neurodegenerative markers unchanged in FCtx proteomic data.

Protein	*p* Value	Log_2_ (FC)
APP	0.592	0
MAPT	0.622	−0.01
SNCA	0.816	0.01
LRRK2	0.737	0.11
PARK7	0.608	−0.09
TH	---	---
TARDBP	0.1	0
GFAP	0.323	−0.14
AIF1L	0.793	0.01

## Data Availability

The datasets used and/or analyzed during the current study and supporting the conclusions of this article are included in this article and Supplementary Materials provided. These datasets are also available from the corresponding author on request. Microbiomic data: http://www.ncbi.nlm.nih.gov/bioproject/1274013 (accessed on 21 May 2025). Proteomic data: https://massive.ucsd.edu/ProteoSAFe/dataset.jsp?accession=MSV000098819 (accessed on 21 May 2025).

## Supplementary Materials

Supporting information can be downloaded at: https://www.mdpi.com/article/10.3390/ijms26178121/s1.

## References

[1] Hou K., Wu Z.X., Chen X.Y., Wang J.Q., Zhang D., Xiao C., Zhu D., Koya J.B., Wei L., Li J. (2022). Microbiota in health and diseases. Signal Transduct. Target. Ther..

[2] Fülling C., Dinan T.G., Cryan J.F. (2019). Gut Microbe to Brain Signaling: What Happens in Vagus…. Neuron.

[3] Sasmita A.O. (2019). Modification of the gut microbiome to combat neurodegeneration. Rev. Neurosci..

[4] McGuinness A.J., Loughman A., Foster J.A., Jacka F. (2024). Mood Disorders: The Gut Bacteriome and Beyond. Biol. Psychiatry.

[5] Thompson S.L., Ellegood J., Bowdish D.M.E., Lerch J.P., Foster J.A. (2024). Sex- and brain region-specific alterations in brain volume in germ-free mice. iScience.

[6] Venkidesh B.S., Narasimhamurthy R.K., Jnana A., Reghunathan D., Sharan K., Chandraguthi S.G., Saigal M., Murali T.S., Mumbrekar K.D. (2023). Pelvic irradiation induces behavioural and neuronal damage through gut dysbiosis in a rat model. Chem.-Biol. Interact..

[7] Horseman T.S., Parajuli B., Frank A.M., Weaver A., Schauer D.A., Moran S., Anderson J.A., Holmes-Hampton G., Burmeister D.M. (2024). Microbiome and Inflammasome Alterations Found During Radiation Dose Finding in a Sinclair Minipig Model of Gastrointestinal Acute Radiation Syndrome. Shock.

[8] Levy R. (2024). The prefrontal cortex: From monkey to man. Brain.

[9] Jones D.T., Graff-Radford J. (2021). Executive Dysfunction and the Prefrontal Cortex. Contin. Lifelong Learn. Neurol..

[10] Szklarczyk D., Gable A.L., Nastou K.C., Lyon D., Kirsch R., Pyysalo S., Doncheva N.T., Legeay M., Fang T., Bork P. (2021). The STRING database in 2021: Customizable protein-protein networks, and functional characterization of user-uploaded gene/measurement sets. Nucleic Acids Res..

[11] Sherman B.T., Hao M., Qiu J., Jiao X., Baseler M.W., Lane H.C., Imamichi T., Chang W. (2022). DAVID: A web server for functional enrichment analysis and functional annotation of gene lists (2021 update). Nucleic Acids Res..

[12] Huang D.W., Sherman B.T., Lempicki R.A. (2009). Systematic and integrative analysis of large gene lists using DAVID bioinformatics resources. Nat. Protoc..

[13] Chen K.W., Demarco B., Broz P. (2020). Pannexin-1 promotes NLRP3 activation during apoptosis but is dispensable for canonical or noncanonical inflammasome activation. Eur. J. Immunol..

[14] Liu C., Shen Y., Huang L., Wang J. (2022). TLR2/caspase-5/Panx1 pathway mediates necrosis-induced NLRP3 inflammasome activation in macrophages during acute kidney injury. Cell Death Discov..

[15] Qin L., Wu X., Block M.L., Liu Y., Breese G.R., Hong J.S., Knapp D.J., Crews F.T. (2007). Systemic LPS causes chronic neuroinflammation and progressive neurodegeneration. Glia.

[16] Cryan J.F., O’Riordan K.J., Cowan C.S., Sandhu K.V., Bastiaanssen T.F., Boehme M., Codagnone M.G., Cussotto S., Fulling C., Golubeva A.V. (2019). The Microbiota-Gut-Brain Axis. Physiol. Rev..

[17] Strandwitz P. (2018). Neurotransmitter modulation by the gut microbiota. Brain Res..

[18] Chen Y., Xu J., Chen Y. (2021). Regulation of Neurotransmitters by the Gut Microbiota and Effects on Cognition in Neurological Disorders. Nutrients.

[19] Banerjee A., Nelson D.E. (2019). How Chlamydia trachomatis conquered gut microbiome-derived antimicrobial compounds and found a new home in the eye. Proc. Natl. Acad. Sci. USA.

[20] Horvath T.D., Ihekweazu F.D., Haidacher S.J., Ruan W., Engevik K.A., Fultz R., Hoch K.M., Luna R.A., Oezguen N., Spinler J.K. (2022). Bacteroides ovatus colonization influences the abundance of intestinal short chain fatty acids and neurotransmitters. iScience.

[21] Mandić A.D., Woting A., Jaenicke T., Sander A., Sabrowski W., Rolle-Kampcyk U., von Bergen M., Blaut M. (2019). Clostridium ramosum regulates enterochromaffin cell development and serotonin release. Sci. Rep..

[22] Socała K., Doboszewska U., Szopa A., Serefko A., Włodarczyk M., Zielińska A., Poleszak E., Fichna J., Wlaź P. (2021). The role of microbiota-gut-brain axis in neuropsychiatric and neurological disorders. Pharmacol. Res..

[23] Hamamah S., Aghazarian A., Nazaryan A., Hajnal A., Covasa M. (2022). Role of Microbiota-Gut-Brain Axis in Regulating Dopaminergic Signaling. Biomedicines.

[24] Dicks L.M.T. (2022). Gut Bacteria and Neurotransmitters. Microorganisms.

[25] Xu L., Wang S., Wu L., Cao H., Fan Y., Wang X., Yu Z., Zhou M., Gao R., Wang J. (2024). Coprococcus eutactus screened from healthy adolescent attenuates chronic restraint stress-induced depression-like changes in adolescent mice: Potential. roles in the microbiome and neurotransmitter modulation. J. Affect. Disord..

[26] Leisner J.J., Laursen B.G., Prévost H., Drider D., Dalgaard P. (2007). Carnobacterium: Positive and negative effects in the environment and in foods. FEMS Microbiol. Rev..

[27] Cuesta S., Burdisso P., Segev A., Kourrich S., Sperandio V. (2022). Gut colonization by Proteobacteria alters host metabolism and modulates cocaine neurobehavioral responses. Cell Host Microbe.

[28] Dagorn A., Chapalain A., Mijouin L., Hillion M., Duclairoir-Poc C., Chevalier S., Taupin L., Orange N., Feuilloley M.G. (2013). Effect of GABA, a bacterial metabolite, on Pseudomonas fluorescens surface properties and cytotoxicity. Int. J. Mol. Sci..

[29] Barandouzi Z.A., Lee J., del Carmen Rosas M., Chen J., Henderson W.A., Starkweather A.R., Cong X.S. (2022). Associations of neurotransmitters and the gut microbiome with emotional distress in mixed type of irritable bowel syndrome. Sci. Rep..

[30] Binda C., Lopetuso L.R., Rizzatti G., Gibiino G., Cennamo V., Gasbarrini A. (2018). Actinobacteria: A relevant minority for the maintenance of gut homeostasis. Dig. Liver Dis..

[31] Zhang Z., Li D., Xie F., Muhetaer G., Zhang H. (2023). The cause-and-effect relationship between gut microbiota abundance and carcinoid syndrome: A bidirectional Mendelian randomization study. Front. Microbiol..

[32] Wickborn K., van der Weijden C.W.J., de Vries E.F.J., Meijer T.W.H., Kramer M.C.A., Spikman J.M., Buunk A.M., van der Hoorn A. (2024). Timeline of cognitive impairments after radiotherapy for head and neck cancer: A review. Clin. Transl. Radiat. Oncol..

[33] Krueger M.E., Boles J.S., Simon Z.D., Alvarez S.D., McFarland N.R., Okun M.S., Zimmermann M.E., Forsmark C.E., Tansey M.G. (2025). Comparative analysis of Parkinson’s and inflammatory bowel disease gut microbiomes reveals shared butyrate-producing bacteria depletion. npj Park. Dis..

[34] Mitrea L., Nemeş S.A., Szabo K., Teleky B.E., Vodnar D.C. (2022). Guts Imbalance Imbalances the Brain: A Review of Gut Microbiota Association With Neurological and Psychiatric Disorders. Front. Med..

[35] Cooke M.B., Catchlove S., Tooley K.L. (2022). Examining the Influence of the Human Gut Microbiota on Cognition and Stress: A Systematic Review of the Literature. Nutrients.

[36] Iacono D., Murphy E.K., Avantsa S.S., Perl D.P., Day R.M. (2021). Reduction of pTau and APP levels in mammalian brain after low-dose radiation. Sci. Rep..

[37] Almond P.R., Biggs P.J., Coursey B.M., Hanson W.F., Huq M.S., Nath R., Rogers D.W. (1999). AAPM’s TG-51 protocol for clinical reference dosimetry of high-energy photon and electron beams. Med. Phys..

[38] Caporaso J.G., Lauber C.L., Walters W.A., Berg-Lyons D., Lozupone C.A., Turnbaugh P.J., Fierer N., Knight R. (2011). Global patterns of 16S rRNA diversity at a depth of millions of sequences per sample. Proc. Natl. Acad. Sci. USA.

[39] Thompson L.R., Sanders J.G., McDonald D., Amir A., Ladau J., Locey K.J., Prill R.J., Tripathi A., Gibbons S.M., Ackermann G. (2017). A communal catalogue reveals Earth’s multiscale microbial diversity. Nature.

[40] Straub D., Blackwell N., Langarica-Fuentes A., Peltzer A., Nahnsen S., Kleindienst S. (2020). Interpretations of Environmental Microbial Community Studies Are Biased by the Selected 16S rRNA (Gene) Amplicon Sequencing Pipeline. Front. Microbiol..

[41] Andrews S. (2010). FastQC: A Quality Control Tool for High Throughput Sequence Data. https://www.bioinformatics.babraham.ac.uk/projects/fastqc/.

[42] Martin M. (2011). Cutadapt removes adapter sequences from high-throughput sequencing reads. EMBnet J..

[43] Callahan B.J., McMurdie P.J., Rosen M.J., Han A.W., Johnson A.J., Holmes S.P. (2016). DADA2: High-resolution sample inference from Illumina amplicon data. Nat. Methods.

[44] Hughes C.S., Moggridge S., Müller T., Sorensen P.H., Morin G.B., Krijgsveld J. (2019). Single-pot, solid-phase-enhanced sample preparation for proteomics experiments. Nat. Protoc..

[45] Wang Y., Yang F., Gritsenko M.A., Wang Y., Clauss T., Liu T., Shen Y., Monroe M.E., Lopez-Ferrer D., Reno T. (2011). Reversed-phase chromatography with multiple fraction concatenation strategy for proteome profiling of human MCF10A cells. Proteomics.

[46] Herbrich S.M., Cole R.N., West K.P., Schulze K., Yager J.D., Groopman J.D., Christian P., Wu L., O’Meally R.N., May D.H. (2013). Statistical inference from multiple iTRAQ experiments without using common reference standards. J. Proteome Res..

