# Supplementary material for: Abscopal Brain Proteomic Changes Associated with Microbiome Alterations Induced by Gastrointestinal Acute Radiation Syndrome in Swine
Source: Int J Mol Sci. 2025 Aug 22;26(17):8121. doi: 10.3390/ijms26178121 (PMC12428343; doi:10.3390/ijms26178121)

Supplemental Figure 1. Summary of Animal Features

Weight, temperature, and condition of swine in RAD and CONTROL groups were tracked daily.

		Weight (kg)						
	**Animal ID**	**Day 0**	**Day 1**	**Day 2**	**Day 3**	**Day 7**	**Day 10**	**Day 14**
**CONTROL**	**25031**	26.6	26.6	27.6	26.1	27.6		
	**25034**	30.15	29.8	29.6	31.3	31.5		
	**25048**	27.2	25.7	25.7	27.2	28.9		
	**25189**	29.5	28.6	28.7	29.9	28.3		
	**25200**	25.85	25.5	25.6	26.6	29.5		
	**25246**	28.3	28.4	28.1	28.9	28.6		
**RAD**	**25197**	26.7	26.9	26.9	27.5	24.5	25.7	25.9
	**25198**	27.4	26.2	26.1	27.7	28.6	27.8	28.2
	**4640**	22.9	22.4	22.5	22	21.6	22.1	20
	**4718**	22.8	23.2	23	23.3	20.4	21.3	20.4
	**4740**	17.5	17.6	18		16.5	17.6	17.9
	**4894**	18.5	17.3	17.9	17.2	17.8	18.3	17.8

		Temperature (C)						
	**Animal ID**	**Day 0**	**Day 1**	**Day 2**	**Day 3**	**Day 7**	**Day 10**	**Day 14**
**CONTROL**	**25031**	36.9	38.6	38.7	37.9	37.5		
	**25034**		37.7	37.7	37.7	37.7		
	**25048**		38.3	38.5	39	37.5		
	**25189**		35	38.8	39.2	38.3		
	**25200**		38.9	38.6	38.5	38.5		
	**25246**	36.9	39.3	39	38.8	38.7		
**RAD**	**25197**		36.1	38.2	37.5	38.3	38.5	37.5
	**25198**		37.8	38.1	38.1	38.2	37.7	37.1
	**4640**		37.6	37.6	37.5	36.9	37.9	35.8
	**4718**	38.6	37.9	38.7	37.7	38.3	37.3	35.6
	**4740**		35.8	37.8	38.2	37.8	36.7	36.4
	**4894**		38.3	38.9	39.2	39.3	38.9	37.5

		Condition						
	**Animal ID**	**Day 0**	**Day 1**	**Day 2**	**Day 3**	**Day 7**	**Day 10**	**Day 14**
**CONTROL**	**25031**	wound redness / swelling						
	**25034**	wound redness / swelling		Diarhea	Eating / playing			
	**25048**	vomiting, wound redness / swelling						
	**25189**			loose stool				
	**25200**	blood in stool, overactive, wound redness / swelling						
	**25246**	wound redness / swelling						
**RAD**	**25197**	vomiting, wound redness / swelling. low energy		diarhea, low energy		reduced appetite		
	**25198**	wound redness / swelling		diarhea	diarhea	reduced appetite		
	**4640**			diarhea	diarhea, decreased appetite, low energy	soft stool		
	**4718**					loose stool	diarhea	
	**4740**			low energy, loose stool, reduced appetite	loose stool			
	**4894**	Vomiting	Soft stool		diarhea, reduced appetite			

Supplemental Figure 2. Gut microbiota community diversity after 8 Gy GI irradiation.
(A) Shannon and Faith’s Phylogenetic alpha diversity (not significant, mean ± SEM). (B) Principle Coordinate Analyses (PCoA) show significant difference in beta diversity determined by Bray-Curtis (q=0.026) but not by generalized UniFrac (q=0.079). RAD n=5, CONTROL n=6

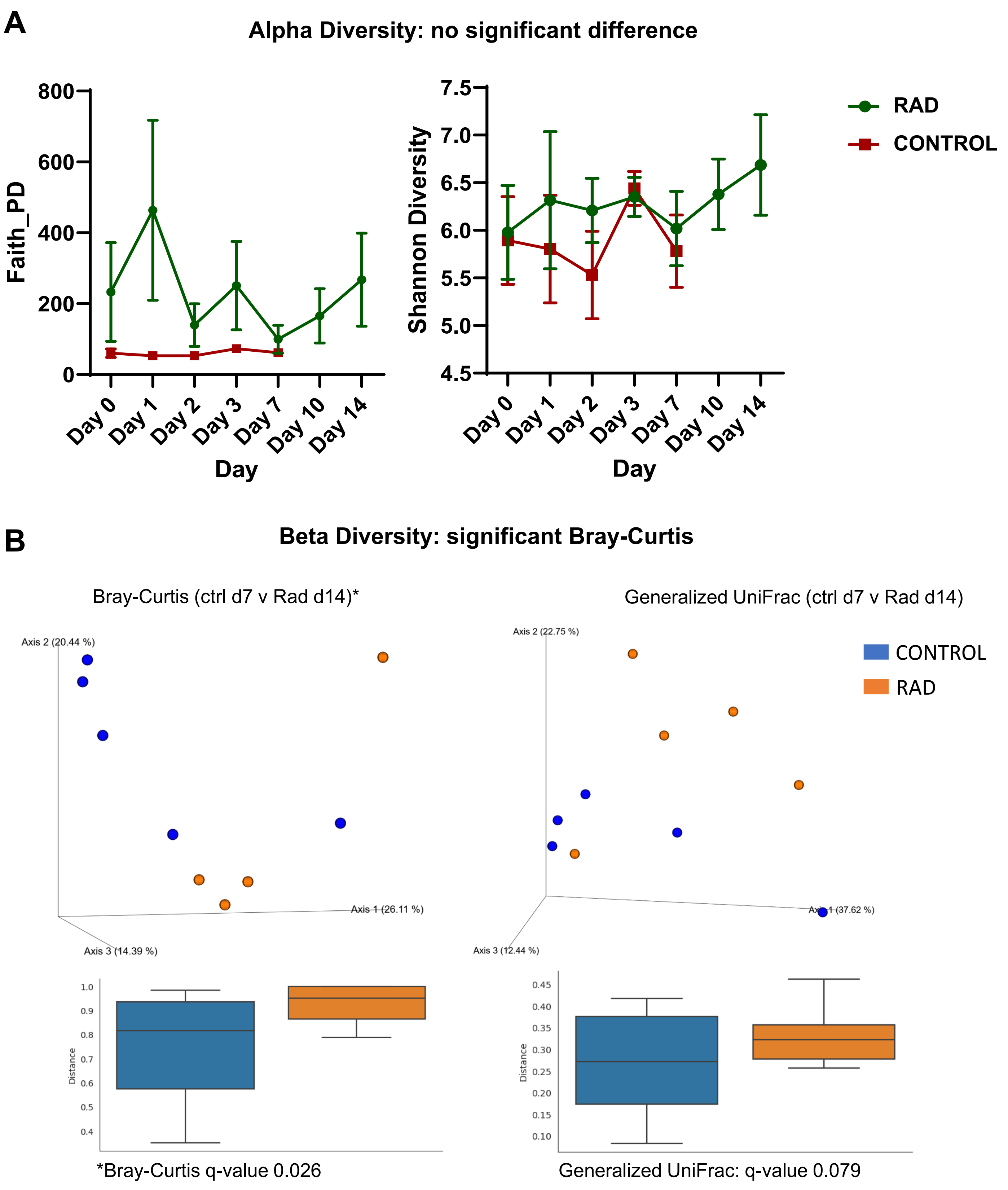

Supplemental Figure 3. Preliminary checks following proteomic analyses in RAD vs. CONTROL swine FCtx. Protein quality and consistency was appropriate for proteomic analysis, as evidenced by the protein abundances across samples (A). PCA of proteomic profiles shows mixed clustering of RAD (orange) vs. CONTROL (blue) FCtx protein expression (B), further demonstrated by the heat map of expression pattern clustering (C), where green indicates low and red indicates high differential protein abundance. Hierarchal clustering of samples. Z-score transformation of normalized protein abundances from a quantitative proteomics analysis using isobaric mass tags was applied before performing the hierarchical clustering based on Euclidean distance and complete (furthest neighbors) linkage. The horizontal dendogram shows the proteins in samples that clustered together. RAD n=6, CONTROL n=6.

CONTROL

RAD

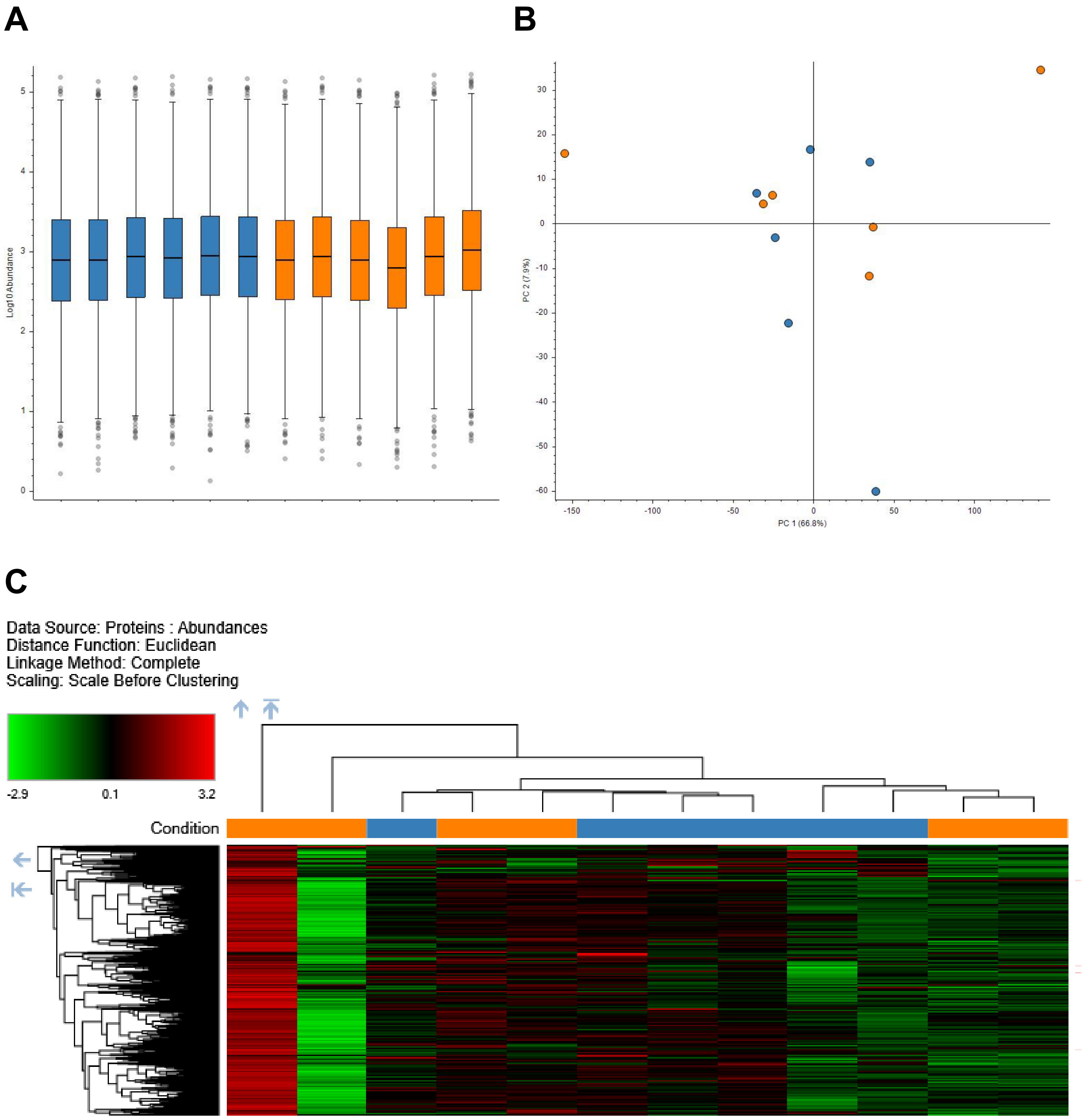

Supplement: Supplementary file 1 [file ijms-26-08121-s001.zip › Supplementary Information.pdf]
